# Small samples and evolution: did the law of small numbers arise as an adaptation to environmental challenges?

**DOI:** 10.3389/fpsyg.2015.00029

**Published:** 2015-02-05

**Authors:** Gorka Navarrete, Carlos Santamaría, Dan Froimovitch

**Affiliations:** ^1^Laboratory of Cognitive and Social Neuroscience, Psychology Department, Universidad Diego Portales, UDP-INECO Foundation Core on NeuroscienceSantiago, Chile; ^2^Cognitive Psychology Department, University of La LagunaTenerife, Spain; ^3^Department of Physiology, University of TorontoToronto, ON, Canada

**Keywords:** gambler's fallacy, fixed probabilities, evolutionary psychology, biases, hot hand belief, law of small numbers

In the context of casino gambling, only a minority (~15%) of players presented with a streak of at least length 6 in roulette disregard recent events in deciding their next move, which is the normatively optimal approach to such a decision (Croson and Sundali, 2005). The majority of people would instead subscribe to a belief in a recency effect. This intriguing pattern of reasoning is categorized as either the *gambler's fallacy*, when the subject perceives negative recency (GF; Laplace, 1951; Tune, 1964; Tversky and Kahneman, 1971), or as the *hot hand fallacy*, when positive recency is perceived (HH; Gilovich et al., 1985). Such tendencies demonstrate, among a variety of things, that magical thinking is not exclusive to astrologists and tarot fanatics. Both the GF and HH refer to instances of the subject projecting a relationship between prior and present events, albeit in opposing directions. For example, subsequent to observing a run of 6 “heads,” a subject committing the GF would expect “tails” on the next coin toss. Alternatively, a subject committing the HH, following a similar streak of, say, successful basketball throws, would expect another “hit” on the next throw. Both fallacies have been posited as consequences of our immanent adherence to the *law of small numbers*—a distorted *conception of chance, according to which short random sequences are considered highly representative of their underlying generating process* (Tversky and Kahneman, 1971; Gilovich et al., 1985); But, counterintuitively, when dealing with sequences governed by chance, the short sub-sequences that we mistake as essentially representative of the overall generating process, actually deviate systematically from sequential properties on the global level; such small sub-sequences, on the basis of which we draw predictive inferences, are rather misrepresentative, containing excessive alternations and lacking sufficient long runs (Gilovich et al., 1985).

When predicting the next outcome in a random bivariate sequence of events, after having observed a local streak in either direction, we tend to fall into one of two behavioral categories, depending on how random the underlying process is perceived to be (Burns and Corpus, 2004). In accordance with the law of small numbers, when the conception of a random generating process is committed to, we expect the next event following a streak of a particular signal to switch to the alternate signal. Alternatively, when the generating process is believed to be nonrandom, we tend to expect the next signal to be consistent with that of the preceding streak. In very simple terms, given a streak in one direction (e.g., three heads in a row):

- When a causal mechanism explaining the streak does not easily come to mind, we tend to commit the GF (e.g., after a few heads, we believe the next throw is more likely to land tails). This occurs most often when the sequential probability is perceived to be fixed (Navarrete and Santamaría, 2012).
- When a causal mechanism is easily accessible (e.g., tampered coin, hot hand, etc.), and the sequence appears to be non-representative of our typified notion of a random sequence, we tend to commit the HH (e.g., after a few successful shots, the player is more likely to succeed again).

In general, we hold—or are inclined to feel as though we hold—a certain degree of control over the events of our immediate environment (Harris and Osman, 2012). We tend to think that the probability of experiencing a car accident is related to our performance behind the wheel; and while this is oftentimes the case, it is definitely not the case as frequently as we would like. The difficulties humans encounter in dealing with phenomena of fixed probabilities are likely related to the fact that, amidst our proximal surroundings, things rarely appear to occur by pure chance. Ordinary events around us are sourced in recognizable causes and elicit appreciable consequences. Moreover, we are innately specialized in discerning patterns (Lopes, 1982) and cause-effect relationships between successive events—especially those in temporal proximity to one another. From an ecological standpoint it is rare for one to observe sequential events that are completely independent of each other (Ayton and Fischer, 2004). Thus, it is of little surprise that we exhibit a distinctive ineptitude when it comes to handling random sequences.

Situations in which past events bear no influence on those of future ones, and, in particular, in which the probability of sequential outcomes is fixed, are primarily confined to games of chance, psychology laboratories, and sample spaces that tend toward infinity (Navarrete and Santamaría, 2012). Games of chance are known to be commonly addictive—a feature perhaps attributable to an illusory sense of control linked to an incapability to understand how they operate. One could argue that games of chance were created with the intent of deceiving humans (Pinker, 1997). Moreover ecological circumstances in which the sample-size accessible to the subject exceeds a few dozen events are virtually absent from a rural or hunter gatherer setting (and likely from any other). Throughout our evolutionary history, it is likely that humans confronted minimally-sized samples exclusively, for which our current limited-capacity numerical cognition served us adequately (as a cautionary side note, see (Navarrete and Santamaría, 2011) for a comment on why such evolutionary arguments should be treated with special care). The numerical representations we seem hardwired to invoke are ill-suited for the processing of large samples. Dehaene et al. propose that we have, in essence, a very precise number-sense for reasoning about very small quantities (~4), and a separate modality—blurred and less precise— that we apply to large quantities (Dehaene, 1986; Feigenson et al., 2004). Evidently, our limited working memory and numerical cognition systems have historically been sufficient for our persistence as a species, and a more sophisticated reasoning apparatus was never selected for. If the inherent restrictions imposed by our numeric reasoning systems are compounded with the known functional constraints on working memory (and those of attention; Hahn and Warren, 2009), a likely outcome would be an inability to maintain accurate mental record of over a few dozen events of a given sample space, and more generally, an inability to work with complex datasets without external aid (i.e., computers, modern statistics, etc.). This might partly explain our erroneous intuitions concerning randomness (Kahneman and Tversky, 1972), and our tendency to expect random sampling to satisfy the law of small numbers (Tversky and Kahneman, 1971; Kahneman and Tversky, 1972).

Limitations on memory and attention in a context where information-access is constrained by design (small samples) renders likely a belief in the law of small numbers. We tend to generalize on the basis of limited samples because this has probably been our only decision-making option throughout our evolutionary history. With the advent and advancement of statistical methodologies and machine learning algorithms, we now know that there are more accurate performing strategies, but the sheer volume of resources required as a substrate for their operation may never have been possible (see (Olsson and Brown, 2010) for an example of how the smartest strategy is not necessarily the better within a foraging context). Prejudices are exemplary of how the use of minimal information to reach far-fetched conclusions may make sense. While morally controversial, prejudices seem to confer an adaptive function. With respect to decision making in the natural world, such generalizations lead to favorable outcomes more often than decisions left up to chance. Furthermore, a strong proclivity toward over-generalization is probably—or once was—critical to survival. If this morning I witnessed a fellow human enter a cave and subsequently get eaten by a bear, it is likely in my interests to be particularly cautious about entering into new caves from now on. Despite the incongruity between the law of small numbers and normative models (Tversky and Kahneman, 1974) the former may be a close-to-optimal system, given the set of possibilities available to the human agent in its ecological setting.

The access to samples of only a limited size throughout our evolutionary history would circumscribe the inferential strategies available to a given individual. An example can be seen in the context of mate selection. The probability of finding a couple possessing a set of specific qualities would depend on the distribution of such traits within the population, and negatively correlated with the number and scarcity of those qualities. The size of the population one has access to determines the nature of successful strategies. In small-to-medium communities or primitive societies, the possibility of being rejected by the few “ideal candidates” warranted a higher degree of flexibility. In modern times, a change in bar, city, or mere patience would suffice. But in the face of minimal information, an individual who considers such as a good representation of all possible cases, and quickly reacts and adjusts to it, would be potentially more efficient than an individual that uses a more resource-hungry approach. When confronting limited opportunities for success, there is incentive to reach a conclusion as efficiently and promptly as possible.

The very fact that there commonly exist causal agents—both tangible and identifiable—that account for the things that happen around us renders the HH (Gilovich et al., 1985) sensible in natural contexts (Pinker, 1997; Wilke and Barrett, 2009). Who wouldn't cede his spear to the hunter that nailed the last 3 in a row over the one that is well known for his accuracy, but has missed the last 3 attempts? We are accustomed to seeking out and characterizing underlying causes of the phenomena we encounter: perhaps the better hunter is feeling unwell, one might speculate. Conversely, it might be conjectured that the hunter who is on fire has been practicing copiously and discovered a new trick. Chance invariably plays an important role; but learning, sickness, and a few hundred other specific and quantifiable causes are also of significance (Ayton and Fischer, 2004). On the other hand, the small role we tend to attribute to chance, let alone pure randomness, and the very little experience we have dealing with it, combined with our habit of treating small samples as representative of the population to which they belong, renders natural the emergence of the GF in non-ecological settings, such as casinos and games of chance. As mentioned, the law of small numbers is likely not the best possible performing strategy with respect to inferential accuracy, but it is saliently conservative in resource utilization, and necessitates very little information to function, as compared to other more sophisticated systems. And in an environment where a great deal of observable events have appreciable underlying causes, a generally rapid-trigger inductive system could be not only good enough, but even a superior alternative to time and resource consuming systems. A similar argument has been made by Gigerenzer and colleagues regarding one-reason decision-making (Gigerenzer and Goldstein, 1996).

Both GF and HH have been historically considered clear and somehow embarrassing fallacies, deriving from our tendency to believe that small samples are representative of the population (law of small numbers). Some authors have recently put forward arguments concerning the notion that the HH may often lead to accurate conclusions regarding the generation of sequential events, and confer evolutionary value (Haselton et al., 2009; Wilke and Barrett, 2009). We believe some of these arguments can also be applied to the gambler's fallacy, and generally to our intrinsic adherence to the law of small numbers. In forming our causal accounts of phenomena, however, we tend to overlook the role of chance—the unexplainable variation or noise that pervades our acquired data. The survival of a particular tribe does not depend on the numeric average of an infinite ideal population, but rather on the hunters returning to the village with meat. And we all know that to arrive at the average necessitates our surrendering at least some of the details that give sense to the world. Human survival profoundly hinges on an uncanny ability to decipher sensible patterns amidst an overwhelming flux of peripheral stimuli, in practice ignoring the chance part of the equation. Although it is an unavoidable truth that we often make mistakes, the fact that we continue to stick around might be living proof that we are right more often than not; so, perhaps the *law of small numbers* is not so bad after all.

## Conflict of interest statement

The authors declare that the research was conducted in the absence of any commercial or financial relationships that could be construed as a potential conflict of interest.
